# Promising for patients or deeply disturbing? The ethical and legal aspects of deepfake therapy

**DOI:** 10.1136/jme-2024-109985

**Published:** 2024-07-09

**Authors:** Saar Hoek, Suzanne Metselaar, Corrette Ploem, Marieke Bak

**Affiliations:** 1 Law Centre for Health and Life, Faculty of Law, University of Amsterdam, Amsterdam, Netherlands; 2 Department of Ethics, Law & Humanities, Amsterdam UMC, Amsterdam, Netherlands; 3 Institute for History and Ethics of Medicine, Technical University of Munich, Munchen, Germany

**Keywords:** Ethics, Law, Mental Health, Information Technology

## Abstract

Deepfakes are hyper-realistic but fabricated videos created with the use of artificial intelligence. In the context of psychotherapy, the first studies on using deepfake technology are emerging, with potential applications including grief counselling and treatment for sexual violence-related trauma. This paper explores these applications from the perspective of medical ethics and health law. First, we question whether deepfake therapy can truly constitute good care. Important risks are dangerous situations or ‘triggers’ to the patient during data collection for the creation of a deepfake, and when deepfake therapy is started, there are risks of overattachment and blurring of reality, which can complicate the grieving process or alter perceptions of perpetrators. Therapists must mitigate these risks, but more research is needed to evaluate deepfake therapy’s efficacy before it can be used at all. Second, we address the implications for the person depicted in the deepfake. We describe how privacy and portrait law apply and argue that the legitimate interests of those receiving therapy should outweigh the interests of the depicted, as long as the therapy is an effective and ‘last resort’ treatment option, overseen by a therapist and the deepfakes are handled carefully. We suggest specific preventative measures that can be taken to protect the depicted person’s privacy. Finally, we call for qualitative research with patients and therapists to explore dependencies and other unintended consequences. In conclusion, while deepfake therapy holds promise, the competing interests and ethicolegal complexities demand careful consideration and further investigation alongside the development and implementation of this technology.

## Introduction

As technology rapidly advances and becomes ever more intertwined with our lives, the line between reality and fabrication can become blurry. This is illustrated by the rise of deepfakes in society. Deepfake technology relies on deep learning, a form of artificial intelligence (AI), to create “hyper-realistic videos digitally manipulated to depict people saying and doing things that never actually happened” (p. 40).^1^ Visual deepfakes work by allowing a person (the ‘source’) to control the facial expressions of another person (the ‘target’) in a (real-time) video, based on large amounts of visual data that the AI model used to learn how to recreate the target’s face. From falsifying speeches by prominent politicians or committing digital identity fraud to creating malicious, non-consenting pornographic content, there are various negative uses of deepfake technology. In addition to direct harms to those portrayed, deepfakes might harm societal trust and diminish the evidential value of video material.^2 3^ Deepfakes can also be used for beneficial social and medical purposes, for instance, deepfaking the faces of relatives so that people with Alzheimer’s disease may keep recognising their ageing loved ones.^4 5^ Other positive examples are celebrities portrayed to speak different languages in international public health campaign videos (like David Beckham in the ‘Malaria Must Die Initiative’^1^) or people with advanced amyotrophic lateral sclerosis (ALS) speaking with their own voice through deepfake voice technology^6^.

Recently, the idea emerged of using deepfakes in psychotherapy, with potential applications including trauma processing and grief counselling (box 1). The very first clinical study in this area used deepfake technology for victims with sexual violence-related post-traumatic stress disorder (PTSD), where deepfakes allowed confronting their perpetrator, whose image is controlled and voiced by the therapist.^7^ PTSD is characterised by intrusive memories, avoidant behaviours and heightened feelings of stress, triggered by trauma-related stimuli.^8^ As PTSD can have a major negative impact on quality of life, effective treatment is key. Using ‘deepfake therapy’ for this purpose may be a promising option. A similar case can be made for grief counselling. The loss of a loved one can have serious implications for one’s mental and social well-being, and people with complicated or prolonged grief may benefit from counselling.^9^ Evidence-based interventions typically include “some form of guided encounter with the memory of the loved one, as in a symbolic monologue or dialogue with the deceased” (p. 355).^9^ This is usually done without technology, but to better help to process grief, virtual interactions with a simulated version of the deceased person may be beneficial.^10 11^

Box 1 **Two therapeutic applications of deepfake technology**

**Sexual violence-related PTSD treatment**

In a recent case report by van Minnen *et al* 7, a novel deepfake therapy platform is described that allowed two women with PTSD due to sexual violence to engage in virtual conversations with a deepfake representation of their perpetrator via Zoom. The deepfake perpetrator was generated by uploading a photograph of the actual perpetrator onto the platform. During the therapy sessions, a therapist trained in treating sexual violence victims with PTSD assumed the role of the perpetrator. Importantly, the therapist did not react as the perpetrator would have done: rather, the therapist consistently responded with empathy to reduce the victim’s self-blame and encourage self-forgiveness. This approach was found to provide a space for the patients to share their traumatic experiences and the resulting distress with the simulated perpetrator. Research into the effectiveness of deepfake therapy in this context has recently started.

**Grief counselling**

A recent South Korean documentary showed a mother interacting with a virtual avatar based on her deceased daughter as a form of exposure therapy for prolonged grief disorder.^10^ Going one step further, deepfake technology could provide a therapeutic space for grieving individuals to engage more realistically in conversations or activities with the virtual representation of their loved ones. In 2020, a Dutch documentary filmmaker brought together computer vision scholars and grief therapists to provide a selected group of people with deepfake grief therapy.^11^ The deepfake therapy sessions were facilitated by a trained therapist who assumed the role of the deceased person or guided the interactions with the virtual representation. By providing support and guidance during the sessions, the therapist could help process emotions of grief, sharing of memories, and expression of unresolved feelings or ‘unfinished business’. Whether the work in this film will be further developed into an evidence-based tool for therapists is not clear. To our knowledge, deepfake therapy for grief counselling is currently not used in mental healthcare practices.

The ethical and legal context and concerns of deepfake therapy have hardly received attention as of yet. Of course, we do not yet know whether the use of deepfakes will become part of mainstream mental healthcare. Right now, deepfakes are introduced in psychotherapy mostly in an experimental context, but this will likely change if their effectiveness is demonstrated. Also, the field of mental health has seen an increase in the use of (commercial) technologies such as chatbots^12^: one day those chatbots might be extended with deepfaked voices and faces. Uncertainty about potential realisation and implementation is intrinsic to emerging technologies, and this does not absolve us from critical ethical reflection.^13^ Rather, it is important to reflect on the promises and risks of emergent technologies ahead of their implementation, before they are ingrained in healthcare and we are in too deep, unable to steer the use of deepfake technology in the right direction. This paper aims to address the current research gap in medical ethics and health law. After having explained how deepfake therapy is supposed to work, we first reflect on whether and how deepfake therapy may constitute good care for the person receiving the therapy, highlighting the implications for the patient and therapist. Second, we discuss the normative implications of deepfake therapy for the person depicted. We show the tension between these different interests and provide recommendations for implementing deepfake therapy responsibly.

## How would deepfake therapy work?

**Figure 1:**
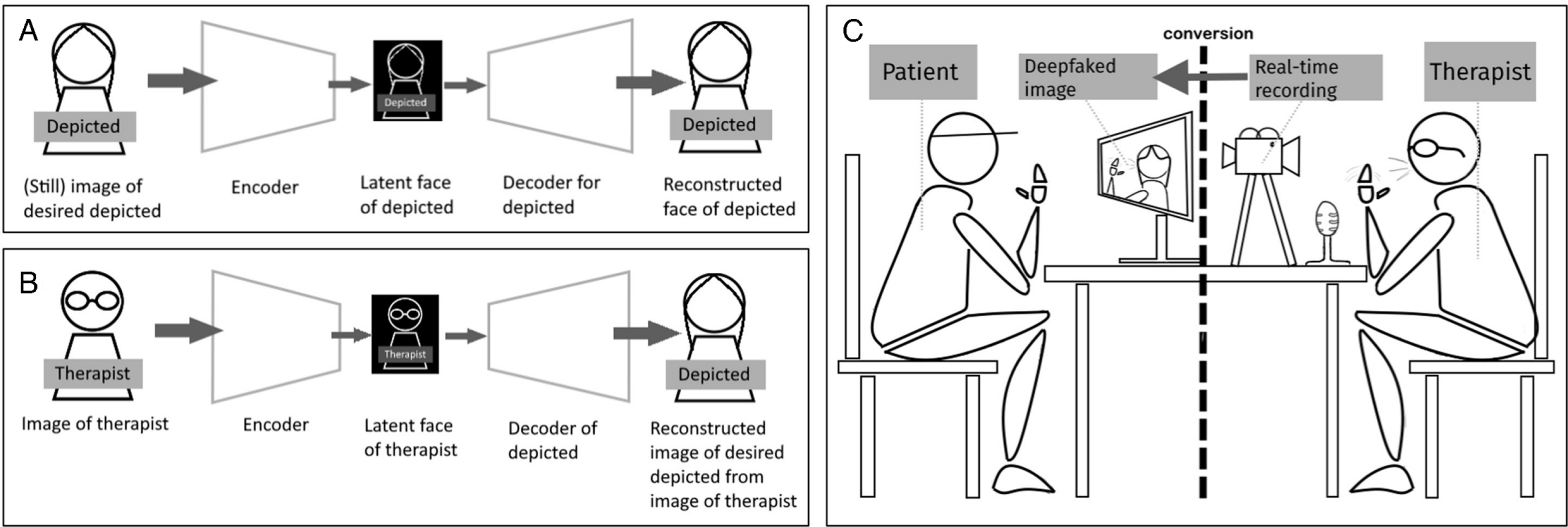
The process of creating a deepfake to be used in psychotherapy. (A) Training: encoder and decoder creation of the depicted; (B) Conversion: reconstruction of the image by using the encoded latent face of the therapist and the decoder of the depicted, to create an image of the depicted than can be controlled by the therapist; (C) Visualisation of a deepfake therapy session where the therapist’s face is converted to the depicted in real time, recording from a different room or a remote location.

The initial step to enable deepfake therapy is training the technology, which revolves around the encoders and decoders that allow face swapping. First, an encoder is used to create what is referred to as a latent face. A latent face is not an image that bears resemblance to an actual face, but rather a set of variables representing facial features, for example, nose shape or eye colour. This will be done for both the desired depicted (ie, the deceased relative or the perpetrator) and the therapist, with the same encoder to ensure compatibility. Then, this latent face can be used by a decoder to reconstruct an image, which is trained only on the image of the person to be depicted. In the generation or conversion phase, this decoder turns the latent face of the therapist into an image of the face of the perpetrator or deceased person. This means, to put it more simply, that a therapist will be recording themselves in a separate room away from the patient, while the recording will—in real time—be encoded into a latent representation of the therapist speaking, which in turn will be converted into the moving image of the desired depicted (mimicking the movements and, at least currently, bearing the voice of the therapist). The patient will see this deepfake virtually, which can be in another room in the same building as the therapist but could also be done remotely. While deepfakes might be employed in a number of different ways in therapy, the process we describe here is the one currently most commonly being tested, and therefore our focus in this article. Figure 1 provides a simplified overview of the deepfake training and conversion processes (figures 1A,B) and a visualisation of the therapy session (figure 1C).

## Can using deepfakes be part of good care?

In the following, we consider how deepfake therapy relates to principles of good care, in relation to the impact on the patient and the moral and legal obligations of therapists deploying the technology. Healthcare is predominantly governed by bioethical principles, national legislation and professional guidelines, and what a therapist has to take into account in terms of quality standards, treatment plans, and general rights and obligations will vary country by country. However, what transcends national jurisdictions is that any form of therapy, including that which uses deepfakes, should qualify as ‘good care’ from a legal as well as an ethical perspective.

### What is good care from a legal and ethical perspective?

Most national legal systems contain the general ‘duty of providing good care’ which also plays an important role in jurisprudence. Fulfilling one’s duty of care in general implies that healthcare providers, when caring for their patients, adhere to the medical-professional standard, including guidelines, protocols, medical ethical codes and the like, established by the profession itself, as well as to health legislation and other documents guaranteeing the rights and interests of the patient involved.^1^ Good care implies that the care provided is of a good quality, that is, safe, effective and efficient and tailored to the patient’s real needs. Binding regulations of the European Union (EU) on medical devices (particularly relevant here is the Medical Device Regulation) and AI (AI Act) include rules that ensure that unsafe, defective or harmful medical devices do not enter medical practice or the market: we find that according to these regulations, deepfakes for therapeutic purposes would classify as medical devices and thus as high-risk AI systems which are subject to stricter requirements. However, it should be noted that a legal framework is nothing without enforcement, which particularly holds for the dynamic field of emerging technologies such as AI.^14^

When introducing deepfake technology in therapy sessions, it is paramount to not only respect legal standards but also the core bioethical principles: respect for autonomy, non-maleficence, beneficence and justice.^15^ These principles are of particular importance in therapeutic settings because of the dependency relationships between care providers, patients and their relatives. First, to respect autonomy, healthcare providers should thoroughly discuss the pros and cons of deepfake therapy with their patients and provide them the opportunity to refrain from it, similar to any treatment option. Second, the therapist must serve the well-being of the patient and limit damage as much as possible: using deepfakes should promote the patient’s physical, psychological and social functioning while not having disproportional or unacceptable physical or psychological side effects. The principle of non-maleficence also implies taking the duty of confidentiality into proper consideration when engaging privacy invasive technology in any therapy (see the section on ‘the rights of the depicted’). Finally, once good care based on deepfake technology is possible, this form of care should be equitably accessible to all patients in need of this type of therapy, unless this would put an unjustifiable burden on the (mental) healthcare system, for instance, in terms of costs.

So, in principle, both legal standards and ethical principles leave room for the use of deepfakes in therapy if it has been proven that their use respects the rights of everyone involved, is safe and has more advantages than disadvantages, does not unnecessarily burden the healthcare system, and is accessible for everyone—once it has been proven that deepfake therapy constitutes good care, not using them might, at some point and for some cases, even be considered negligent and at odds with the professional standard. Currently, the problem is that the effectiveness of deepfake therapy has not been validated and benefits and risks are not yet clear. Yet also when these benefits and risks of deepfake therapy in different settings are clear, this does not automatically mean that the therapist may offer such therapy as ‘good care’ to patients. Namely, it is required that the therapy’s benefits outweigh the harms (proportionality) and that it involves the least intrusive alternative (subsidiarity) compared with similar technologies such as virtual reality therapy, imagery rehearsal and traditional exposure therapy.^8^ Whether deepfake therapy has benefits over these—and other—forms of therapy requires further research. As long as it is unclear whether deepfake therapy meets not only the aforementioned standards and principles for good care but also the criteria of proportionality and subsidiarity, such therapy should be proposed only to patients in a clinical research context. In terms of possible risks of deepfake therapy, we anticipate several potential psychological harms that require special attention, which we describe hereafter using our two cases.

### Specific risks of deepfake therapy: overattachment and the blurring of reality

Potential harms to the patient may begin to arise in the process of collecting data for the creation of deepfakes, which largely relies on what the patient brings along. If these data are not readily available, patients should not be placed—especially in the sexual violence case—in dangerous situations in order to collect videos or photographs. Even if the data are available, the retrieval of photographs from personal archives or social media may be a ‘triggering’ moment for a patient: it may cause emotional distress as it arouses feelings or memories associated with the trauma. This risk is not specific to deepfake therapy as it can also be invoked by other forms of grief counselling, but it should nonetheless be taken into account and discussed with the patient before a decision is made about using deepfake therapy. What is new about deepfake therapy, is that people might start to curate video content of their living loved ones specifically for the purpose of future grief counselling, which can positively or negatively impact those relationships (cf.^16^ who discuss this point in relation to chatbots of reincarnated loved ones).

Then, once deepfake therapy is started, we think that the simulated confrontation with the subject of a person’s trauma may cause specific risks related to dependencies and a blurring of reality. First, the patient–therapist relationship itself may be impacted given that the therapist is controlling the deepfake: the patient may start to associate or even identify the therapist with the perpetrator or deceased loved one. This could possibly lead to confusion, feelings of unsafety or an unhealthy attachment to the therapist. Second, a patient could become ‘addicted’ to communicating with the generated image. This is most likely to happen in the grief counselling case. In the Dutch documentary, a woman about to speak with the deepfake of her deceased husband, noted that she had felt like ‘going on a date with him’, and worried that she would want to do that more often after the therapy session. This is reminiscent of an episode of the sci-fi series Black Mirror (Episode ‘Be Right Back’), in which a woman reincarnates her boyfriend first through a chatbot based on his digital footprint, and finally as a lifelike robot. Research on chatbots has already reported accounts of real individuals coping with grief who have become attached to chatbots, created through apps like Paradot or Replika, acting as their lost loved one.^17 18^ However, as opposed to chatbots that operate without being controlled by a human, which can have disastrous consequences^2^, deepfake therapy should function, at least for now, in a safe and controlled therapeutic setting where the therapist is in control of what is said by the deepfake. Still, in the (near?) future, the existing ‘griefbots’ might be supplemented with deepfaked images of the deceased. This would exacerbate existing risks of therapeutic deepfakes and create new ethical concerns: in particular, concerns about financial motives by the companies developing those griefbots, who might purposefully aim at creating attachments and dependencies to keep (paying) users engaged; and who might sell data about personal conversations to data brokers.^16 19^

Although chatbots are currently separate from deepfakes, both technologies simulate interpersonal interactions and could potentially interfere with an important element of grief: accepting the reality of one’s loss. This may in turn lead to what is called ‘complicated grief’. One aspect of complicated grief is the excessive avoidance of reminders of the loss, and the inability to comprehend the finality of the loss.^20^ This is why many forms of grief therapy focus on acceptance of loss.^21^ While using deepfakes in therapy may potentially help with accepting the loss (e.g. by having a farewell conversation with the deepfake), the use of deepfakes may also reinforce the feeling that the deceased person is somehow still there, and obstruct full acceptance of loss. While outright deception is not so much a concern for deepfake technology in psychotherapy as it is in other settings, like politics, the line between what is real and not might still become blurred. Although patients rationally know the deepfake they are interacting with is not a real person, on an emotional, transactional level, it may feel very real. This could have serious drawbacks, not only for the grief counselling case where deepfake therapy might undermine the authentic relation with the deceased. Could victims of sexual trauma, for example, regain a misplaced and perhaps dangerous semblance of trust towards the perpetrator of their trauma? The therapist should monitor and try to mitigate these risks of attachment and blurring of reality, which may require a different approach according to each patient’s personal characteristics—for instance, whether someone is prone to addiction—and grieving style, and also differs by type and extent of use.

## The rights of the depicted: healing through stealing?

Deepfakes are often generated without consent. This use of a natural person’s image without their consent raises the question of whether there are legal and ethical grounds to object to such use within the context of therapy. We first describe the legal background and then discuss the ethical-legal aspects involved in balancing benefit for the patient who undergoes deepfake therapy with the rights of the depicted whose image is used (or stolen?). By ‘the depicted’ we mean the perpetrator and the deceased person, respectively, in the cases of sexual violence-related PTSD treatment and grief counselling.

### Data protection and portrait rights

In the EU, the use of one’s image or likeness is regulated by the General Data Protection Regulation (GDPR), which finds its roots in the fundamental principles of privacy and personal autonomy.^3^ The GDPR emphasises the importance of obtaining explicit and freely given consent in relation to the processing of personal data, especially when it concerns sensitive data.^4^ As the creation of deepfakes requires the collection of personal data (e.g. images from social media platforms) and because the deepfake itself is designed to possess identifiability as a key characteristic, they unmistakably qualify as a form of personal data. However, this does not automatically mean that the depicted person may object to therapeutic use of their deepfaked image. In the case of grief counselling, the depicted person is deceased and the GDPR does not apply to the personal data of deceased persons (Recital 27), and therefore, cannot be called on for objecting. Member States can still provide for additional postmortem privacy protection in their national implementation of the GDPR, and in their national health laws, but this is not harmonised across the EU and only some countries give next-of-kin the right to consent to the deceased person’s data being processed.^22^ The USA, similarly, has no federal laws extending to postmortem privacy; only state laws.^23^ In addition to data protection legislation, some jurisdictions protect so-called portrait rights (e.g. the Netherlands) or personality rights (e.g. the USA) intended to safeguard an individual’s image or likeness, which may also grant limited postmortem protection rights to the deceased person’s relatives. This protection is primarily intended to prevent commercial use of their image or likeness without consent, as opposed to personal use. This is similar to the GDPR which does not apply to data processed only for personal or household activities.^5^ However, providing deepfake therapy cannot be seen as the latter, since it, clearly, comes down to using someone’s picture in a professional therapy setting. Thus, the creation of a therapeutic deepfake should be done in compliance with those laws.

### Balancing therapeutic benefit with privacy

What does this mean for our two cases? Regarding the grief counselling case, despite the lack of clear and harmonised legal rules, one of the authors has previously argued for a moral right to postmortem privacy (albeit one that may be outweighed by the interests of living individuals).^24^ Deepfakes are morally problematic if the deepfaked person would object to the way in which they are represented,^2^ and we find that this is still the case when the depicted is deceased because it goes against the wishes of the person who continues to exist as ‘informational entity’. If the deceased has explicitly stated an objection to deepfake therapy while alive (which might become the case in the future when this therapy is more well known) it would be unethical and potentially illegal to still use their image. Doing so would also harm societal trust in psychotherapists if this became publicly known. When the person has not objected while alive, or their preference is unknown to surviving relatives, we find that deepfake therapy is morally permissible if there are clear benefits to the patient. Whenever possible, for instance, in the case of terminal illness, it would be most respectful to ask for consent prospectively while the person to be depicted as a deepfake is still alive (some authors have even suggested the creation of digital ‘do not resurrect’ orders^16^). Potentially, the person undergoing deepfake therapy should discuss this with other living family members too, who might take offence to deepfake therapy due to worries about the distortion of their loved one’s image or the instrumentalisation itself.

Regarding the PTSD therapy case, when we consider a living perpetrator of sexual violence, data processing should be based on consent, which is unlikely to be given, or another valid legal basis such as the ‘legitimate interest’ of the therapist and their patient. How to balance the rights of the non-consenting deepfaked perpetrator against the patient’s legitimate interest in therapy? One could say that the perpetrator’s likeness is simply used as a means to an end: their simulation might be saying things they do not agree with.^2^ In cases of data breach, the deepfake may also cause ‘reputational injury’ to the perpetrator, as it impacts their social identity and reputation when a deepfake video is seen and believed by others.^25^ It could also be asserted that there is no legitimate interest of the patient, as described in the GDPR^6^; that the processing of data is not strictly ‘necessary’ in this context, as alternative therapeutic methods are available. On the other hand, as van Minnen *et al* 7 state, the deepfake method primarily targets patients who have exhausted conventional therapeutic approaches relying on imagination or the use of photographs. Thus, if deepfake therapy can be beneficial for sexual-violence-related PTSD victims, as a last resort for a considerable health problem, we find that using a deepfake without consent would be acceptable in light of the legitimate interest of the patient. Especially so because the risks of data breaches are minimal in a therapy setting, and various protective measures can be taken, similar to how other confidential information in mental healthcare is protected. This consideration is in line with a survey among the general public which finds that deepfake (voice) transformations are more accepted when they are used inside a therapeutic context.^26^

Specific measures should be taken in both cases to protect the depicted person’s privacy. First, the deepfake and any personal data used for its creation should be stored locally and securely. Second, given that therapists are unlikely to be directly involved in the deepfake creation process, it is imperative to carefully vet and select a third party with expertise in medical or therapeutic applications, and a comprehensive understanding of privacy concerns and cybersecurity. Similarly, as deepfakes are a digital medium, the patient will interact with them through videocall software, which should be secure and therapy-specific, rather than relying on general-use commercial companies such as Zoom or Teams, which can be less transparent about their data storage, monitoring and use.^7^ Standard contractual clauses would be helpful for drafting agreements with technology companies creating deepfakes and proving the videocall software. Third, therapists should ensure that in cases of a data breach it remains clear that the video is fake, for instance, by always using their own voice rather than a deepfaked voice, or by incorporating resilient watermarks in the deepfake. If such precautionary measures are taken, and importantly, the deepfake therapy is a good and more effective alternative to ‘regular’ therapy (see the ‘Can using deepfakes be part of good care’ section), it is likely that the legitimate interests of the therapist and the patient outweigh any privacy concerns the depicted person may have. Still, their interests, as well as any broader societal concerns, should be taken into consideration. In box 2, we list some points to consider before using deepfake therapy. This list is not exhaustive and may need to be updated once deepfakes are actually used in care.

Box 2 **Considerations for the responsible use of deepfake therapy**

- **Quality of care:** Demonstrated effectiveness of the therapy, that outweighs the disadvantages, and compliance with quality and safety standards, are ethical prerequisites for using deepfake therapy in clinical settings.
- **Consideration of alternatives:** Less intrusive (exposure) therapies, both in terms of risks to the patient and privacy concerns of the depicted, should be considered and exhausted before using deepfake therapy, at least until there is more clarity about benefits and harms.
- **Oversight and control by the therapist:** Deepfake therapy should be conducted in real time by a licensed therapist voicing the depicted. If applications are developed where the deepfake can be used at home by the patient or is even coupled with an AI-based chatbot, clear rules should be set up to safeguard responsible use in unsupervised home settings.
- **Consent and privacy of the depicted:** In principle, consent from the depicted needs to be obtained for the creation of a deepfake. In cases where consent is impracticable, for instance, in the case of depicting a perpetrator, the legitimate interest of the patient suffices, although coupled with appropriate data security and governance measures to protect the privacy and portrait of the depicted. These may include using watermarks or the therapist’s own voice in the video, to mark it as a deepfake, as well as good contractual agreements with providers of the deepfake technology and video platform.
- **Societal concerns:** Deepfake therapy should be societally accepted before implementing it into practice, and this may require public dialogue; broader fairness concerns such as equal access to deepfake therapy and the environmental impact, should be considered before deciding whether deepfakes should be introduced in mental healthcare.

## Concluding remarks

This paper is the first to reflect on the normative aspects of deepfake therapy, a potentially promising new technology that may also have deeply disturbing implications in terms of dependency and privacy. We discussed two cases that raise several ethical and legal concerns, although our descriptions of these applications remained necessarily general, because the technology is not (yet) employed in standard mental healthcare practice. To ascertain whether deepfake therapy can constitute good care, more ethical, legal, social and psychological research is needed into its effects, merits and drawbacks. Clinical evaluation studies should be subject to ethical and legal review that considers the potential harms to participants as well as the interests of those depicted by deepfake technology, both of which we explored in this paper. We argued that the legitimate interests of those who would benefit from deepfake therapy should outweigh the interest of the depicted as long as the deepfakes are handled carefully and discreetly. We suggest that qualitative research with patients and therapists should be conducted to explore the risk of overattachment and potential other unintended consequences for persons receiving deepfake therapy. Moreover, further guidance will be needed on the specific responsibilities of therapists and on how deepfake technology in mental healthcare can fulfil the criteria of the EU’s new AI Act, which likely classifies this type of AI system as a high-risk system. Developments are fast and ongoing interdisciplinary normative reflection is needed, especially if chatbots were to be involved in deepfake therapy, which would exacerbate the potential risks that we identified and create new ethicolegal concerns.

## Data Availability

No data are available.

## Footnotes

Article 4—professional standards—of the Convention for the Protection of Human Rights and Dignity of the Human Being with regard to the Application of Biology and Medicine: Convention on Human Rights and Biomedicine, Oviedo 1997.

https://www.vice.com/en/article/pkadgm/man-dies-by-suicide-after-talking-with-ai-chatbot-widow-says

See Art. 1 GDPR as well as Art. 5–11 GDPR.

See Art. 5 and Art. 7 GDPR.

See Art. 2(c) GDPR.

Art. 6(f) of the GDPR

See: Lomas, N. (9 August 2023). *Zoom knots itself a legal tangle over use of customer data for training AI models*. (https://techcrunch.com/2023/08/08/zoom-data-mining-for-ai-terms-gdpr-eprivacy/?guccounter=1). See also: Dutch Government (16 February 2022). *Public DPIA Teams OneDrive SharePoint and Azure AD*. (https://www.rijksoverheid.nl/documenten/publicaties/2022/02/21/public-dpia-teams-onedrive-sharepoint-and-azure-ad).
